# Evaluation of Pediatric Patients’ General Health Status Prior to Dental Treatment Under General Anesthesia: A Retrospective Study

**DOI:** 10.3390/children12070903

**Published:** 2025-07-08

**Authors:** Enes Bardakçı, Şemsettin Yıldız, Betül Yazmacı, Mehmet Emin Doğan, Kübra Mumcu, Mehmet Sinan Doğan

**Affiliations:** 1 Department of Pediatric Dentistry, Faculty of Dentistry, Harran University, Sanliurfa 63050, Turkey; enesbardakc67@harran.edu.tr (E.B.); betulyazmaci@harran.edu.tr (B.Y.); dt.kubramumcu@harran.edu.tr (K.M.); 2 Department of Pediatric Dentistry, Faculty of Dentistry, Gaziantep University, Gaziantep 27410, Turkey; semsettin_yildiz@hotmail.com; 3 Department of Dentomaxillofacial Radiology, Faculty of Dentistry, Harran University, Sanliurfa 63200, Turkey; medogan@harran.edu.tr

**Keywords:** general anesthesia, pediatric dentistry, dental treatment, health status, uncooperative child

## Abstract

**Background/Aim**: Dental treatment is typically performed under general anesthesia for children who have difficulty cooperating, as well as for those with mental or physical disabilities requiring special care. This study aims to categorize and evaluate the systemic disease or syndrome status, age, and gender of children who require dental treatment under general anesthesia. **Materials and Methods**: In this study, the ages, sexes, disabilities (if any), and systemic diseases of patients requiring dental treatment under general anesthesia (GA) between the ages of 1 and 15 were analyzed. The patients were categorized based on having difficulties cooperating or having mental or physical disabilities that require special care. **Results**: In this study, data from 1666 patients were examined. A total of 955 patients (57.32%) were male, while 711 (42.67%) were female. Overall, 232 (13.9%) patients with disabilities or systemic diseases visited the clinic, including 49 who had epilepsy (2.9%), the highest number among the systemic disease group. This finding was statistically significant in the 4–6 age group (*p* < 0.00). **Conclusions**: Among patients with special needs, epilepsy emerged as a disorder that requires the most dental treatment. We believe that providing dental treatment for children with neurological diseases, such as epilepsy, in a fully equipped operating room will be beneficial in managing complications that may arise during treatment.

## 1. Introduction

Chronic and progressive oral diseases can affect individuals throughout their lives, and dental caries is one of the most common non-communicable and chronic diseases [1,2].

Early childhood caries (ECC) is one of the most common health problems among children. It presents with many unique clinical features, including the rapid development of caries that affect multiple teeth shortly after they emerge in the oral cavity. These lesions affect the tooth surfaces that are less susceptible to the development of caries. ECC is a multifactorial disease resulting from the interaction between several factors, including cariogenic microorganisms, exposure to fermentable carbohydrates due to poor nutritional practices, and various social variables. ECC is a severe health condition that occurs among children living in socially disadvantaged communities, where malnutrition leads to social and health inequalities. Because children with early childhood caries are young and often experience dental anxiety, when dental treatment cannot be performed in a chair with basic behavioral management techniques, it may need to be conducted under general anesthesia (GA) [3,4,5].

Decayed teeth can cause functional (chewing, speaking, biting, etc.), psychological (e.g., loss of self-confidence), and economic problems. Untreated cavities may result in painful sensations and the risk of developing sepsis. In addition, they affect the quality of life of children and their families. Oral health is integral to overall health, well-being, and quality of life, and most oral diseases can be prevented or treated in their early stages [2,6].

Behavioral guidance techniques for pediatric patients constitute an essential part of pediatric dentistry. As a result, it is advised to perform dental treatment in children under local anesthesia [7,8].

However, for healthy children who are unable to cooperate and who have additional systemic diseases or particular disabilities, treatment must be performed under general anesthesia or sedation [8,9]. Similarly to the variety of indications for anesthesia during dental interventions, patients’ existing systemic diseases and anesthesia practices also exhibit diverse characteristics. It is known that some drugs used in the treatment of systemic diseases, especially respiratory tract disorders, have indirect effects on oral health. Inhaled corticosteroids, which are widely used in the treatment of asthma, are reported to disrupt the balance of the oral microbiota by changing the composition of the saliva, and this situation has negative effects on oral and periodontal health [10]. Although most dental procedures, such as dental fillings and extractions, can be performed under local anesthesia, general anesthesia is often required due to incompatibility. This is especially true for pediatric patients, including those who have difficulty cooperating due to intellectual disabilities, psychiatric disorders, severe anxiety, advanced craniofacial anomalies, and orofacial trauma [11,12]. This patient group comprises patients who require special healthcare. Early childhood caries is seen in these patients due to difficulties in cooperation and inadequate oral hygiene, and dental treatments usually cannot be planned all at once and comprehensively. In addition, since these patients cannot maintain oral care, the success rate with conservative approaches decreases, and this makes tooth extractions inevitable. For all of these reasons, dental treatments are performed under general anesthesia in these patient groups [13].

Pediatric patients must undergo several examinations to determine whether dental treatment can be performed under general anesthesia [14,15]. According to the latest literature published by the American Academy of Pediatric Dentistry (AAPD), dental treatment under general anesthesia is recommended for children requiring multiple dental procedures who are unable to cooperate with behavioral guidance techniques, as well as for those with mental, physical, or systemic disorders that hinder treatment [14,16].

To apply the correct treatments and increase efficiency in dental treatment for children who are uncooperative and have intellectual disabilities, as well as in complex dental therapies for individuals with systemic diseases (especially in cases where close monitoring is required, such as hypertension, diabetes, and epilepsy), general anesthesia is indicated for maxillofacial surgeries requiring major intervention; for patients with a phobia of dental practices; and for those unable to undergo the procedure due to vomiting/gagging reflexes. General anesthesia is also indicated when local anesthesia would not suffice due to the duration of the treatment, or in cases considered more advantageous in terms of time and cost, such as procedures that require more than one appointment [17].

The disadvantages of general anesthesia are that it requires an experienced anesthesia team and more equipment than treatments performed with local anesthesia, is expensive due to these increased equipment and personnel requirements, and carries risks of mortality and complications in patients with appropriate indications. However, it is a preferred application because it allows dental procedures to be completed in a single session, does not require the cooperation of the patient for treatment success, and increases the quality of life of patients [15,18].

With the application of general anesthesia, depression occurs in sensitive and motor areas throughout the body. As a result, pain is alleviated and muscle relaxation and a loss of consciousness are induced. Thus, the patient does not experience pain during the operation, allowing surgical and dental treatment to be performed easily due to muscle relaxation. There are many differences between pediatric and adult patients regarding general anesthesia. Age, weight, and height vary between these groups. The basal metabolic activity rates differ between children and adults, with this activity being higher in pediatric patients. Because the respiratory rate of children is higher than that of adult patients, great care is taken when administering drugs that suppress the respiratory system [15].

Postoperative symptoms are the most crucial factors to consider after dental treatment is performed under general anesthesia and sedation. Parents should be informed about possible complications that may develop following treatment, with postoperative pain being the most common. Sleep disturbances and restlessness, which may occur at night after treatment, are among the more common symptoms. The patient’s age, medical history, dental treatment, premedication, the anesthetic drugs used, and the duration of anesthesia all influence the frequency of these symptoms [16,19].

The duration of dental rehabilitation varies depending on the complexity of the operation and the type of treatment applied. The average treatment duration varies between 1 and 4 h, and extractions are usually completed in a shorter time compared to restorative procedures. Dental treatments performed under general anesthesia are notable for being able to be safely performed in a single session in a hospital environment. This method is advantageous, especially in pediatric patients, because it does not require cooperation and provides effective pain control. It is also reported to provide higher quality and more durable results than traditional methods [20]. During general anesthesia, complications may occur, including allergic reactions, bronchospasms, nausea with vomiting, pharyngitis, lip swelling, fever, sore throat, and delayed or prolonged recovery. However, it remains a preferred practice because it allows dental treatments to be completed in a single appointment, does not require the patient’s cooperation for success, and enhances the quality of life of patients [21].

This study aims to categorize and evaluate the systemic disease or syndrome status, age, and gender of children who require dental treatment under general anesthesia.

## 2. Materials and Methods

This study was conducted by retrospectively reviewing the clinical records of 1666 patients aged 1–15 years (mean age 4.98) who were treated under general anesthesia at the Pediatric Dentistry Clinic of Harran University Faculty of Dentistry in Şanlıurfa province in the Southeastern Anatolia region of Türkiye. During the treatment process, intraoral, extraoral, physical, systemic, and mental examinations of the patients were performed. The health status of the patients was recorded by listening to their medical history in accordance with the statements of their parents. In addition, decisions were made according to the physical and mental status examinations. In terms of dental treatment, during the first examination, it was decided that the patients could not undergo dental treatment according to the Frankl scale. Among the patients who were definitely negative or negative according to the Frankl scale, patients who were deemed suitable for general anesthesia were selected and the necessary information was recorded. Routine blood tests (hemogram) and additional tests when necessary were requested from patients who underwent general dental anesthesia and were directed to the anesthesiology and reanimation clinic for consultation. Routine blood tests requested for general anesthesia and medical conditions were consulted by medical doctors according to the patients’ histories if necessary.

All patients with data from the general anesthesia registry between December 2017 and May 2022 were included in this study. Ethical committee approval for the study was obtained from the Harran University Clinical Research Ethics Board Commission (HRÜ/22.14.12). The principles of the World Medical Association Declaration of Helsinki were followed in this study. Consent forms obtained from the legal representatives of the patients for the application of general anesthesia and dental treatments are available in the archive. Based on the information in the general anesthesia registry and epicrisis reports of the treated patients, the patients’ age, sex, disabilities, if any, and systemic diseases were analyzed.

## 3. Statistical Analysis

The SPSS (Statistical Package for the Social Sciences Inc., Chicago, IL, USA) 22.0 package program was used to analyze the data. The number and percentage values were used to describe the data using descriptive statistical analyses. The chi-square test was used to compare the significance levels, and the Kolmogorov–Smirnov test was used to examine the data distribution.

## 4. Results

**Figure 1:**
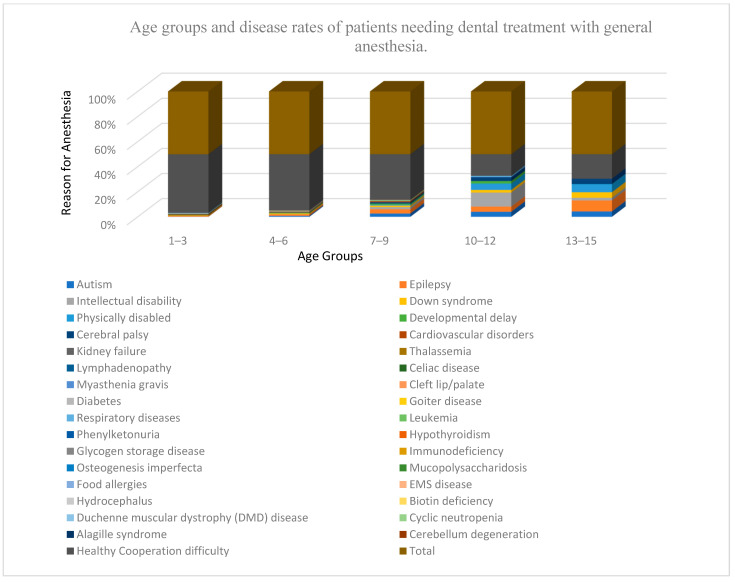
Age groups and disease rates of patients needing dental treatment with general anesthesia.

The data from 1666 patients aged between 1 and 15 years were examined during the study period. Of these patients, 955 were male (57.32%) and 711 were female (42.67%) (Table 1). A total of 1434 (86.1%) patients requiring general anesthesia (GA) were non-cooperative and had no systemic diseases or disabilities. Thus, it was not possible to establish healthy communication using behavioral guidance techniques during examination or treatment. The number of patients visiting the clinic who had disabilities or systemic illnesses was 232 (13.9%), among which 49 had epilepsy (2.9%), the highest number in the systemic illness group (Table 2). Among the patients receiving GA, 957 (57.44%) were in the 4–6 age group, representing the group that most frequently visited the clinic (Table 2, Figure 1). This frequency was significantly higher in the 4–6 age group and among non-cooperative patients (*p* < 0.001).

The highest number of patients receiving GA per year was observed in 2021, at 378 (26.95%), whereas the number of patients receiving GA in 2020 was 98 (5.88%), as shown in Table 1.

## 5. Discussion

In pediatric dentistry, behavioral guidance techniques help clinicians to treat children afraid of dental practices by providing ways to reduce their anxiety and stress levels, make them feel safe, and successfully complete their treatments by collaborating with the child in the clinical environment. However, dental treatments are performed under general anesthesia for children and adolescents who cannot communicate, are incompatible, are extremely fearful, or cannot cooperate with behavioral guidance techniques due to their psychological or emotional development level, as well as their mental, physical, and medical conditions [8,15].

In dental procedures, general anesthesia is regularly performed using modern techniques. It is frequently preferred for patients with systemic diseases or those who cannot undergo dental treatment with local anesthesia for psychological reasons, as it enables multiple treatments to be performed in a single session. Thus, long-term interventions can be safely conducted [9,17,22].

As a fundamental element of general anesthesia, anesthesiologists are primarily responsible for effective and safe airway management throughout the perioperative process. The responsibility for airway traumas, including dental injuries, that may occur during this period also lies with anesthesiologists. Direct laryngoscopy is still the most common method for visualizing the airway during tracheal intubation. However, since dental procedures are performed in the oral cavity, nasal endotracheal intubation is often preferred in these cases where general anesthesia is applied. Although direct laryngoscopy is thought to be significantly associated with dental injuries in difficult airway cases, differences have been reported in the timing and causes of perioperative dental injuries after the development of various airway management tools and the adoption of preventive approaches. In addition, airway problems associated with central nervous system diseases increase the risk of complications during and after the anesthesia process in outpatients. In this patient group, the probability of difficult ventilation and intubation is increased due to skeletal and muscular anomalies in the head and neck region, and appropriate precautions should be taken before anesthesia [9,23].

General anesthesia applications are frequently preferred in complex interventions in oral and dental surgery and may increase the risk of infection development in the postoperative period. In such cases, suppression of the immune system and disruption of the balance in the oral flora may pave the way for the recolonization of pathogenic microorganisms. In this context, the use of probiotics to prevent postoperative infections is noteworthy. Probiotic microorganisms can both suppress pathogens and support the healing process by creating a symbiotic balance in the oral microbiota. Therefore, the application of probiotics as a complementary treatment in the period after general anesthesia offers a strategic approach in protecting periodontal health [24].

Untreated extensive tooth decay in children can lead to acute and chronic infections, pain, psychological discomfort, sleep disturbances, behavioral changes, loss of appetite, and weight loss. Comprehensive dental treatment performed under general anesthesia positively affects oral and dental health, body development, social and psychological well-being, and quality of life, especially in young children. It has also been found to positively affect the family [25].

In our study, the results of dental treatment performed under general anesthesia in healthy pediatric patients aged 1–15 and those with a systemic disease or disability were evaluated retrospectively. In this study, 57.32% of the patients were male and 42.67% were female. In a 2021 study conducted by El Hachem et al. [26], these rates were 44.30% for girls and 55.70% for boys, similar to our findings. In another study, 54.30% of patients were male and 45.70% were female [16]. In another study, 54.2% of patients were female and 45.8% were male [27]. 

Children aged 3–6 experienced issues related to communication and cooperation while in the dental chair due to anxiety and fear. For this reason, it has been reported that dental treatments performed under general anesthesia have increased over the last ten years, especially among patients in the 3–6 age group [15]. Another study found that the average age of patients requiring dental treatment under general anesthesia was 58.8% under six years of age [26]. This study determined that 82.28% of the patients requiring general anesthesia were under 6 years of age. Non-cooperative patients were statistically significantly more likely to undergo treatment under general anesthesia compared to other patients (*p* < 0.001).

A study by Gezgin O. in 2022 reported that 73.50% of patients who received dental treatment under general anesthesia exhibited issues related to cooperation, while 26.50% had systemic disorders or disabilities that prevented treatment [16]. In another study, 41.2% of patients requiring general anesthesia were non-cooperative and 36.1% had medically risky conditions [26]. In another study, 82% of patients receiving GA were non-cooperative [28]. In this study, 86.1% of the patients were non-cooperative and 13.9% had a systemic disease or disability.

General anesthesia is frequently preferred for patients who cannot undergo dental treatment with local anesthesia owing to mental or psychological reasons, as it allows multiple treatments to be performed in a single session, enabling long-term interventions to be performed safely. Completing treatments under general anesthesia in a single session and in a short timeframe for children with special needs provides significant comfort and advantages for both families and children [21].

A previous study reported that autism was the most common condition observed in treated patients (31%) [29]. In another study, 15.4% of children with special needs had encephalopathy, while 13% had autism [30]. Another study found that the two most common conditions were intellectual disability (22.35%) and autism/atypical autism (18.18%) [16]. In this study, among the patients treated under general anesthesia, 24.65% had epilepsy and 12.33% had a physical disability.

Childhood epilepsy is a major health problem on a global scale. Overall, 1 in 150 children will experience epilepsy in their first year of life. However, this rate varies greatly by region: in the United States, approximately 450,000 children are diagnosed with epilepsy under the age of 17. In Canada, the rate is 8.1 per 1000 births, while in Africa, this rate is maintained at 17.3. In some regions, such as Sardinia, the rate is even lower, at 2.67 per 1000 children. These observations may be related to socioeconomic conditions, access to health services, and the options offered. In Iran, prevalence rates of 2.3% have been observed, as well as higher prevalence rates. This necessitates the development of region-specific public health services. In our study, we attribute the prevalence of epilepsy in our cohort to the high spending, growth, and low socioeconomic status of our patients [31,32].

It has been determined that dentist visits decreased because people were confined to their homes due to the COVID-19 pandemic [16,33]. This study supports this situation when examined trends over the years. While the highest number of patients receiving GA was reported in 2021, the number of patients receiving GA was lowest in 2020. We believe that this was due to the effects of the COVID-19 pandemic. We observe a similar situation in the study conducted in 2023. The decrease in clinical activity in patients requiring special care due to the COVID-19 pandemic and the home confinement protocol due to the risks of hospital infection are also seen in their study. They state that they returned to normal activities in the subsequent years [34].

As with other studies, the limitation of our study is that it was based on patient data [29,35,36]. Our clinic is one of the most significant centers providing GA services in the region, and it serves a broad population as patients from the surrounding area apply for its services. Thus, this study sheds light on the need for dental treatment under GA in society.

## 6. Conclusions

Dental treatment under general anesthesia offers significant advantages in pediatric dentistry, particularly for children with special needs and those with difficulty cooperating. Completing all dental procedures in a single session enhances comfort and the overall treatment experience for this patient group. In our study, epilepsy was identified as the most common condition among patients with special needs requiring dental treatment, with a higher prevalence observed among male patients. Furthermore, it was concluded that performing dental treatment on children with neurological disorders, such as epilepsy, in a fully equipped operating room is crucial for effectively managing potential complications. It is thought that more equipment and a fully equipped hospital are needed to meet the need for general anesthesia locally, and that patients and their relatives require further education.

## Figures and Tables

**Table 1:** Gender distribution of patients requiring dental treatment under general anesthesia by year.

Year	Male (n)	Male (%)	Female (n)	Female (%)	Total (%)
2017	69	52.67	62	47.32	7.86
2018	177	58.80	124	41.19	18.06
2019	229	60.58	149	39.41	22.69
2020	53	54.08	45	45.91	5.88
2021	257	57.23	192	42.76	26.95
2022	170	55.01	139	44.98	18.55
Total	**955**	**57.32**	**711**	**42.67**	**100**

**Table 2:** Age groups and disease rates of patients needing dental treatment with general anesthesia.

	Age Groups					
Reason for Anesthesia	1–3	4–6	7–9	10–12	13–15	*p*-Value
Autism	1 (0.1%)	16 (1.0%)	12 (0.7%)	4 (0.2%)	2 (0.1%)	*p* = 0.000
Epilepsy	5 (0.3%)	21 (1.3%)	15 (0.9%)	4 (0.2%)	4 (0.2%)	*p* = 0.000
Intellectual disability	0 (0.0%)	2 (0.1%)	8 (0.5%)	11 (0.7%)	1 (0.1%)	*p* = 0.000
Down syndrome	3 (0.2%)	12 (0.7%)	5 (0.3%)	2 (0.1%)	2 (0.1%)	*p* = 0.000
Physically disabled	0 (0.0%)	3 (0.2%)	3 (0.2%)	5 (0.3%)	3 (0.2%)	*p* = 0.000
Developmental delay	1 (0.1%)	8 (0.5%)	4 (0.2%)	2 (0.1%)	0 (0.0%)	*p* = 0.000
Cerebral palsy	1 (0.1%)	4 (0.2%)	5 (0.3%)	3 (0.2%)	2 (0.1%)	*p* = 0.000
Cardiovascular disorders	3 (0.2%)	5 (0.3%)	4 (0.2%)	0 (0.0%)	0 (0.0%)	*p* = 0.000
Kidney failure	1 (0.1%)	3 (0.2%)	0 (0.0%)	0 (0.0%)	0 (0.0%)	*p* = 0.000
Thalassemia	1 (0.1%)	2 (0.1%)	0 (0.0%)	0 (0.0%)	0 (0.0%)	*p* = 0.000
Lymphadenopathy	1 (0.1%)	0 (0.0%)	0 (0.0%)	0 (0.0%)	0 (0.0%)	*p* = 0.000
Celiac disease	2 (0.1%)	0 (0.0%)	0 (0.0%)	0 (0.0%)	0 (0.0%)	*p* = 0.000
Myasthenia gravis	0 (0.0%)	1 (0.1%)	0 (0.0%)	0 (0.0%)	0 (0.0%)	*p* = 0.000
Cleft lip/palate	1 (0.1%)	1 (0.1%)	0 (0.0%)	0 (0.0%)	0 (0.0%)	*p* = 0.000
Diabetes	0 (0.0%)	1 (0.1%)	0 (0.0%)	0 (0.0%)	0 (0.0%)	*p* = 0.000
Goiter disease	1 (0.1%)	1 (0.1%)	0 (0.0%)	0 (0.0%)	0 (0.0%)	*p* = 0.000
Respiratory diseases	4 (0.2%)	3 (0.2%)	1 (0.1%)	1 (0.1%)	0 (0.0%)	*p* = 0.000
Leukemia	0 (0.0%)	0 (0.0%)	2 (0.1%)	0 (0.0%)	0 (0.0%)	*p* = 0.000
Phenylketonuria	1 (0.1%)	1 (0.1%)	0 (0.0%)	0 (0.0%)	0 (0.0%)	*p* = 0.000
Hypothyroidism	0 (0.0%)	2 (0.1%)	0 (0.0%)	0 (0.0%)	0 (0.0%)	*p* = 0.000
Glycogen storage disease	1 (0.1%)	1 (0.1%)	0 (0.0%)	0 (0.0%)	0 (0.0%)	*p* = 0.000
Immunodeficiency	0 (0.0%)	1 (0.1%)	0 (0.0%)	0 (0.0%)	0 (0.0%)	*p* = 0.000
Osteogenesis imperfecta	0 (0.0%)	1 (0.1%)	0 (0.0%)	0 (0.0%)	0 (0.0%)	*p* = 0.000
Mucopolysaccharidosis	0 (0.0%)	1 (0.1%)	0 (0.0%)	0 (0.0%)	0 (0.0%)	*p* = 0.000
Food allergies	0 (0.0%)	1 (0.1%)	0 (0.0%)	0 (0.0%)	0 (0.0%)	*p* = 0.000
EMS disease	0 (0.0%)	0 (0.0%)	1 (0.1%)	0 (0.0%)	0 (0.0%)	*p* = 0.000
Hydrocephalus	0 (0.0%)	2 (0.1%)	0 (0.0%)	0 (0.0%)	0 (0.0%)	*p* = 0.000
Biotin deficiency	0 (0.0%)	1 (0.1%)	0 (0.0%)	0 (0.0%)	0 (0.0%)	*p* = 0.000
Duchenne muscular dystrophy (DMD) disease	0 (0.0%)	1 (0.1%)	0 (0.0%)	0 (0.0%)	0 (0.0%)	*p* = 0.000
Cyclic neutropenia	0 (0.0%)	1 (0.1%)	0 (0.0%)	0 (0.0%)	0 (0.0%)	*p* = 0.000
Alagille syndrome	0 (0.0%)	1 (0.1%)	0 (0.0%)	0 (0.0%)	0 (0.0%)	*p* = 0.000
Cerebellum degeneration	0 (0.0%)	1 (0.1%)	0 (0.0%)	0 (0.0%)	0 (0.0%)	*p* = 0.000
Healthy Cooperation difficulty	386 (23.2%)	859 (51.6%)	163 (9.8%)	17 (1.0%)	9 (0.5%)	*p* = 0.000
Total	414 (24.84%)	957 (57.44%)	223 (13.38%)	49 (2.94%)	23 (1.38%)	

The rate was significantly higher in the 4–6 age group and non-cooperative patients (*p* < 0.001).

## Data Availability

The data presented are available upon request from the corresponding author.
